# Metabolites and metabolism in vascular calcification: links between adenosine signaling and the methionine cycle

**DOI:** 10.1152/ajpheart.00267.2024

**Published:** 2024-10-25

**Authors:** Parya Behzadi, Cynthia St. Hilaire

**Affiliations:** ^1^Division of Cardiology, Department of Medicine, Pittsburgh Heart, Lung, and Blood Vascular Medicine Institute, University of Pittsburgh, Pittsburgh, Pennsylvania, United States; ^2^Department of Bioengineering, University of Pittsburgh, Pittsburgh, Pennsylvania, United States

**Keywords:** adenosine, arginine methylation, methionine cycle, peripheral artery disease, vascular calcification

## Abstract

The global population of individuals with cardiovascular disease is expanding, and a key risk factor for major adverse cardiovascular events is vascular calcification. The pathogenesis of cardiovascular calcification is complex and multifaceted, with external cues driving epigenetic, transcriptional, and metabolic changes that promote vascular calcification. This review provides an overview of some of the lesser understood molecular processes involved in vascular calcification and discusses the links between calcification pathogenesis and aspects of adenosine signaling and the methionine pathway; the latter of which salvages the essential amino acid methionine, but also provides the substrate critical for methylation, a modification that regulates the function and activity of DNA and proteins. We explore the complex and dynamic nature of osteogenic reprogramming underlying intimal atherosclerotic calcification and medial arterial calcification (MAC). Atherosclerotic calcification is more widely studied; however, emerging studies now show that MAC is a significant pathology independent from atherosclerosis. Furthermore, we emphasize metabolite and metabolic-modulating factors that influence vascular calcification pathogenesis. Although the contributions of these mechanisms are more well-define in relation to atherosclerotic intimal calcification, understanding these pathways may provide crucial mechanistic insights into MAC and inform future therapeutic approaches. Herein, we highlight the significance of adenosine and methyltransferase pathways as key regulators of vascular calcification pathogenesis.

## INTRODUCTION

Vascular calcification in the intimal or medial layer of arteries is a strong risk factor predicting major adverse cardiovascular events. Although intimal calcification occurs in the necrotic core of atherosclerotic plaques, medial arterial calcification (MAC) is the deposition of mineral in the extracellular matrix surrounding vascular smooth muscle cells (SMCs) and is a leading cause of arterial stiffness and triggers thrombosis, which can occlude lower extremity arteries (1). The molecular processes underlying vascular cell osteogenic reprogramming and mineral nucleation are complex, dynamic, and are not fully understood (2). Although external cues such as lipids, extracellular matrix (ECM) breakdown, and the inflammatory signals are well-known disease-driving culprits, additional factors modulate and contribute to disease pathogenesis.

The influence of adenosine in regulating the cardiovascular system—specifically attenuating atrial fibrillation and inducing arterial dilation—has been known since 1929 (3). Adenosine can bind to adenosine receptors or enter the cells via transporters; inside the cell, it can feed into the methionine cycle and other metabolic processes (4). Depletion of intracellular adenosine levels can inhibit methionine methyltransferases activity (5), which metabolize *S*-adenosyl-l-methionine (SAM) to *S*-adenosylhomocysteine (SAH) and elevated levels of SAH feedback to inhibit methyltransferase activity. Methylation of the amino acid arginine is catalyzed by protein arginine methyltransferases (PRMTs), which transfer a methyl group from SAM to the arginine residues, and protein arginine methyltransferases (PRMTs) have been found to be a key regulator of bone and vascular calcification (6–9). This review summarizes the current understanding of these metabolites in the pathogenesis of vascular calcification and the clinical and molecular implications of methyltransferase activity in cardiovascular disease.

## VASCULAR CALCIFICATION: INITIATING MECHANISMS AND CONSEQUENCES

**Figure 1.**
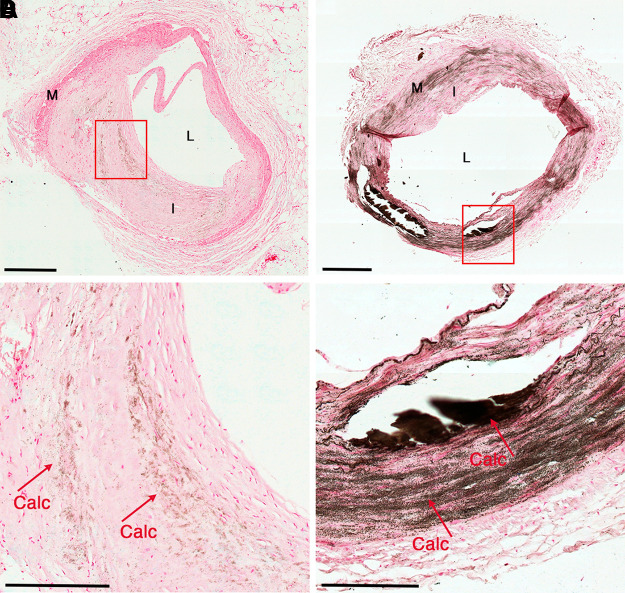
Histological image of calcified vascular tissue. *A*: a human coronary artery with atherosclerosis plaque exhibiting calcification in the neointima. *B*: a human tibial artery exhibiting extensive medial calcification. *C*: magnified views of the boxed region in *A* exhibiting macrocalcification (Calc). *D*: magnified views of the boxed region in *B*, which has severe calcification (Calc). Mineral is stained black by Von Kossa, original magnification ×20 (scale bars: 0.5 mm). I, intima; L, lumen; M, media layer.

The structural integrity of the vessel wall is essential for maintaining vascular homeostasis. In arteries, endothelial cells are exposed to the lumen and sit atop the internal elastic lamina to maintain the barrier between the blood and vessel wall. SMCs are sandwiched between the internal and external elastic lamina and intermingled with proteins that make up the extracellular matrix (ECM) (10). This medial layer of arteries maintains vascular tone and blood flow distribution through the body via the dynamic contraction and relaxation of SMCs and the elasticity of the ECM (10). Arterial stiffening is a major risk factor for the development of cardiovascular disease and is a consequence of loss of elasticity (e.g., breakdown of the elastic lamina, loss of SMCs contractility, calcification) in the vessel wall (11). Vascular calcification manifests in two types: intimal calcification, typically associated with atherosclerosis, and medial calcification, found in the medial layer along elastic fibers (12) (Fig. 1). The accumulation of lipids and inflammatory cells in the arterial wall are initiating stresses that contribute to the formation of atherosclerosis, and calcification is a byproduct and marker of advanced plaque formation (13). In plaques, macrocalcification is observable via computed, positron emission tomography, and even radiological imaging and is associated with so-called “stable plaques” (14). Microcalcifications, falling below the resolution of current imaging modalities, in close proximity to one another are thought to contribute to plaque rupture by increasing the local stress exerted on the plaque (15–17) (Fig. 1, *A* and *C*). Independent from mechanisms driving atherosclerosis pathogenesis, MAC is associated with the disruption of mineral homeostasis in the medial layer of the arteries, primarily affecting SMCs, and is highly prevalent in patients with chronic kidney disease, diabetes, and peripheral artery disease (PAD) (1, 18).

### Atherosclerotic-Specific Mechanisms of Calcification

**Figure 2.**
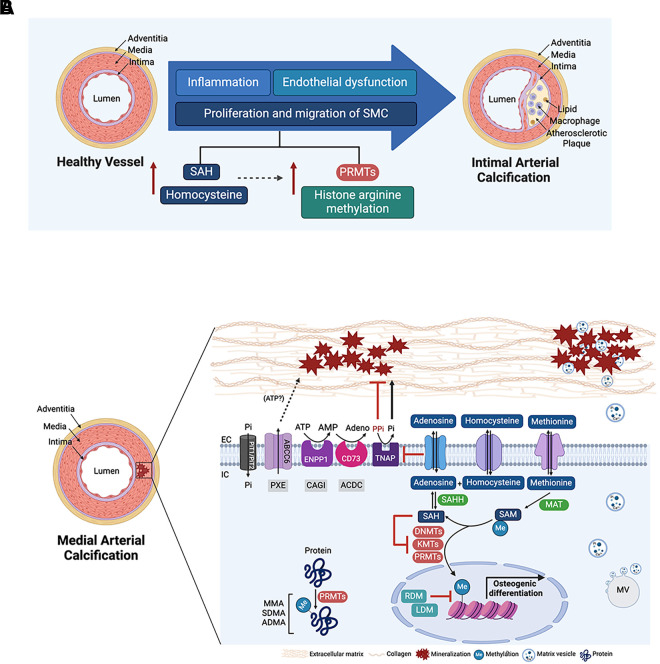
Vascular calcification and methyltransferase activity. *A*: schematic representation of the transition from healthy vessel to intimal arterial calcification in an atherosclerotic plaque. Elevated levels of homocysteine and *S*-adenosylhomocysteine (SAH) promote, endothelial dysfunction, smooth muscle cell (SMC) migration, and proliferation. These processes are linked to the dysregulation of histone arginine methylation and protein arginine methyltransferases (PRMTs) activity. *B*: schematic representation of mechanisms underlying medial arterial calcification. Calcium (Ca^2+^) and inorganic phosphate (Pi) deposition, primarily as hydroxyapatite, occur in the extracellular matrix (EC). ENPP-1 metabolizes extracellular ATP into AMP. Tissue nonspecific alkaline phosphatase (TNAP) converts pyrophosphate (PPi), the endogenous mineralization inhibitor, into the mineral building block, Pi. ATP is recognized as a likely substrate for ABCC6; however, the exact molecule its transport remains unidentified. Adenosine has dual roles, inhibiting TNAP or participating in the methionine cycle and activating methyltransferase activity. Elevated homocysteine and SAH levels contribute to vascular calcification by activating methyltransferase activity and transferring methyl groups from *S*-adenosylmethionine (SAM) through PRMTs to histone arginine residues, to histone lysine residues by methyltransferases (KMTs), and to DNA cysteine by DNA methyltransferases (DNMTs). PRMTs are also found on cytoplasmic proteins. Histone methyltransferase activity is typically inhibited by arginine demethylases (RDMs) and lysine demethylases (LDM), respectively. Pseudoxanthoma elasticum (PXE), generalized arterial calcification of infancy (GACI), and arterial calcification due to CD73 deficiency (ACDC) are rare genetic diseases associated with abnormal medial calcification processes, which result from mutations in a variety of transporter genes. ATP, adenosine triphosphate; AMP, adenosine monophosphate; ENPP-1, ectonucleotide pyrophosphatase/phosphodiesterase 1. Images created with a licensed version of BioRender.com.

Low-density lipoprotein accumulation in the intima initiates atherosclerotic plaque formation, where their lipid contents undergo modifications such as oxidation and aggregation and adversely impact the cells of the vascular wall of large arteries (19). The oxidized lipid activates endothelial cells and SMCs to produce adhesion molecules like vascular cell adhesion molecule-1 (VCAM-1) and intercellular adhesion molecule-1 (ICAM-1), as well as chemokines such as monocyte chemoattractant protein-1 (MCP-1), growth factors like macrophage colony-stimulating factor (M-CSF) and granulocyte-macrophage colony-stimulating factor (GM-CSF) (19–22). These substances interact with receptors on monocytes, promoting their recruitment, transmigration, and differentiation into macrophages. In atherosclerotic plaques, the signaling cascades downstream of macrophage-derived cytokines and growth factors drive the loss of SMC contractility and help to induce osteogenic reprogramming of SMCs (23, 24). Macrophage-derived extracellular vesicles also contribute to microcalcification in human atherosclerotic plaques, where they are abundant and associated with cholesterol crystals (25) (Fig. 2*A*). Extracellular vesicles are equipped with matrix metalloproteases that degrade and remodel ECM while adhering to ECM components (e.g., collagens and glycosaminoglycans) through their integrins and annexins (25, 26). In adult aorta, SMCs are quiescent unless they are injured or stimulated. With the loss of their contractile phenotype, SMCs can transdifferentiate into adipocyte-like, chondrocyte-like, and osteoblast-like cells (27). Regarding osteogenic reprogramming, elegant lineage tracing studies have found that Krüppel-like factor 4 (KLF4)-positive SMCs in atherosclerotic plaques are prone to acquire osteoblast-like calcifying properties (28). With the loss of contractility, SMCs in atherosclerotic plaques migrate and proliferate and help to form a fibrous cap surrounding the lipid-rich core. As plaques develop, an avascular necrotic core forms, and cellular bodies and debris help to induce calcification nucleation (29). As the plaque remodels, the thinning of the fibrous cap coupled with disturbed stress forces caused by microcalcification contributes to the development of unstable and ruptured plaques (30). Microcalcification deposited in part via extracellular vesicles could contribute to plaque destabilization, potentially by altering the texture of the connective stroma within plaque fibrous caps (31). Runt-related transcription factor 2 (*RUNX2*) expression can be stimulated by the reactive oxygen species (ROS) and inflammatory signaling found in atherosclerotic plaques (32–34).

### MAC-Specific Mechanisms of Vascular Calcification

Atherosclerosis primarily affects large arteries, such as the coronary and carotid arteries, while medial arterial calcification (MAC) can occur in any vascular bed, including small and medium-sized arteries, MAC can be found in any vascular bed (1). MAC increases with age, often presenting in the fourth decade of life, initially appearing in abdominal aorta and pelvic arteries, and later spreading proximally to the descending thoracic aorta and aortic arch (35), suggesting that distinct vascular beds have different propensities to calcify. MAC accumulates along damaged elastin fibers in the ECM (Fig. 1, *B* and *D*); however, whether damage to the elastin fibers is a driver or is a consequence of MAC accumulation is still not entirely clear (36, 37). A study involving 718 patients investigated whether there is a specific risk for vascular disease associated with intimal calcification and MAC (38). They found femoral calcification in 77% of participants, with 38% exhibiting intimal arterial calcification and 28% exhibiting MAC. Risk factors for intimal calcification included age, male sex, and smoking, whereas MAC was associated with diabetes and a high ankle-brachial index, and higher levels of circulating inactive Matrix Gla-Protein (MGP) (38).

MAC and nuclear DNA damage accumulate with age and correlate with many disease states (39, 40), and several lines of evidence support the hypothesis that DNA damage and activation of the DNA damage response pathway promote vascular calcification. A recent case study highlights an extreme example of extensive nonatherosclerotic calcification of the aortic root and arch of a 49-yr-old male who at 9 yr of age was treated with 45-Gy mediastinal (sternum) radiation for lymphoma (41). Radiation-induced fibrosis is a common side effect of external radiation therapy, characterized by collagen deposition, reduced vascularity, and bone necrosis (42, 43). Cell death, particularly apoptosis and necroptosis, is associated with mineral nucleation, as it creates a favorable environment for calcium phosphate deposition, analogous to the processes observed in vascular calcification (44). PolyADP-ribose polymerase1 (PARP1) is expressed in response to DNA damage and oxidative stress and functions to aid in DNA break repair (45). PARP1 creates poly (ADP-ribose) (PAR) moieties that are secreted and serve as nidus for calcification (46), and PARylation of the osteogenic master transcription factor, RUNX2, promotes osteogenic gene transcription (47). Mutations underlying Hutchinson-Gilford Progeria syndrome cause chromatin instability and double strand DNA breaks, which activate the mechanosensing DNA damage response, leading to accelerated SMC aging, loss of contractility, and promotion of calcification (48–51). Discoidin domain receptor-1 (DDR1), a collagen-binding receptor tyrosine kinase, is a pivotal mechanosensor that simulates actin stress fiber formation and helps to maintain the cytoskeleton structure. DDR1 senses stiffness surrounding the cell and promotes SMCs calcification via upregulating *RUNX2* expression (52, 53). Deletion of DDR1 disrupts cytoskeleton microtubule formation, and alterations to the cellular structure alone also perturb the nuclear localization of RUNX2 (54).

In mineral nucleation, calcium-rich extracellular vesicles are deposited into and accumulate among the ECM proteins. This serves as a substrate for inorganic phosphate (Pi) binding, enabling the precipitation of calcium phosphate in the ECM during the early stages of MAC pathogenesis. This process occurs before the formation of amorphous calcium phosphate, which later serves as a precursor to the nucleation and growth of hydroxyapatite crystals within the ECM (55, 56). This process involves a disruption in the balance of calcium and phosphate and the loss of inhibitors and represents a compensatory response to vascular calcification (57, 58). The formation and stabilization of hydroxyapatite crystals further help to promote the transition of SMCs to a bone-forming phenotype. SMCs localized to areas of hydroxyapatite show decreased expression of smooth muscle 22α (*SM22α*) and increased expression of bone morphogenetic protein 2 (*BMP-2*), a member of the TGF-β superfamily (59). The osteogenic reprogramming of SMCs is characterized by the expression of *RUNX2* and elevated levels of other key osteogenic markers, including osteopontin (OPN) and osterix (OSX) proteins (60–62). Osteogenic SMCs initiate and propagate MAC by releasing extracellular vesicles, which act as nucleation sites for hydroxyapatite crystal formation (63). Extracellular vesicles released from osteogenic SMCs are enriched in tissue nonspecific alkaline phosphatase (TNAP), encoded by the alkaline phosphatase (*ALPL*) gene, and ectonucleotide pyrophosphatase/phosphodiesterase 1 (ENPP1), which are key enzymes for mineralization (64). ENPP-1 metabolizes extracellular adenosine triphosphate (ATP) to adenosine monophosphate (AMP) and pyrophosphate (PPi), the latter a potent endogenous mineralization inhibitor that acts by binding to the surface of calcium phosphate and prevents the formation of hydroxyapatite crystals (65, 66). Promineralizing TNAP converts the PPi to inorganic phosphate (Pi) to favor the proliferation of hydroxyapatite crystal formation outside the matrix vesicle along collagen fibrils (64), and elevated TNAP contributes to both common and genetic forms of MAC (12, 67–69). Caveolin-1 (CAV1) is involved in the release of calcifying extracellular vesicles1, and it has recently been found that epidermal growth factor receptor (EGFR) inhibition can prevent MAC by reducing CAV1 release (63).

In addition to PPi, fetuin-A, a circulating glycoprotein, and MGP are two proteins involved in the regulation of ECM mineralization in MAC (70, 71). MGP, which is naturally expressed in SMCs, prevents mineralization by binding to hydroxyapatite crystals and promoting their absorption by local macrophages (70). MGP is thought to also regulate SMC osteogenic reprogramming by binding to BMP-2, thereby preventing BMP-2 from activating its receptors and subsequently upregulating RUNX2 (72, 73). MGP knockout mice develop MAC and die prematurely from heart failure (70). Increased collagen accumulation and fragmentation of elastin fibers were observed in the medial layer of arteries in MGP-deficient mice, a phenomenon associated with elevated *Runx2* expression (74, 75). Behind calcium phosphate imbalance, oxidative stress, proliferation, leptin, autophagy, apoptosis, DNA damage response, and high levels of glucose can initiate ECM mineralization and increase the expression of osteogenic markers (57, 76, 77). Hyperglycemia triggers the phosphorylation of AKT proliferation pathway via O-linked *N*-acetylglucosamine, which is associated with an increased expression of *Runx2* and SMCs differentiation (78). Leptin promotes MAC formation in the lower extremity arteries of individuals with type 2 diabetes mellitus by elevating the levels of BMP2 and RUNX2 proteins via the PI3K/Akt signaling pathway (79). In bovine SMCs, leptin induces osteogenic reprogramming and downregulates MGP by modulating the Wnt signaling pathway, enhancing GSK-3β phosphorylation, and increasing TNAP activity (80).

Autophagy appears to have conflicting roles in vascular calcification depending on experimental conditions. In vitro, directly stimulating autophagy via valproic acid treatment protected against phosphate-induced SMCs calcification, whereas inhibition of autophagy in response to Pi led to increase extracellular vesicles release in bovine SMCs (81). A separate in vivo study found that high Pi exhibit upregulation of autophagy markers LC3 and increased mitochondrial ROS generation (82). In vitro studies show that apoptosis alone is sufficient to induce calcification in human SMCs, whereas inhibition of apoptosis can decrease calcification (29, 83).

Although the initiating stresses driving intimal calcification and MAC are distinct, there are many commonalities in how the buildup of mineral occurs in these pathologies (summarized in Fig. 2). It is also important to note that research into the pathogenesis of vascular calcification faces significant challenges due to the difficulty in pinpointing the precise conditions under which calcium and Pi precipitate within the vascular wall. Some in vitro experimental models primarily focus on homogeneous precipitation—e.g., experiments using exogenous Pi supplementation, as opposed to osteogenic differentiation medium, which is the base medium used to differentiate mesenchymal stem cells into osteoblasts with dexamethasone (84)—which occurs only under local supersaturation conditions that do not accurately represent in vivo environments. These experimental conditions may not be fully reflective of the actual pathological state and may skew research outcomes. As a result, some of the prevailing hypotheses regarding the mechanisms of calcification are based on models and concepts that may not accurately mimic real-life scenarios, highlighting a critical limitation in the field. Although these models are not perfect, they still offer valuable insights. However, as a field, we should strive to develop more physiologically relevant in vitro disease models.

## INSIGHTS ON MAC FROM GENETIC DISEASE

A handful of monogenetic diseases exhibit MAC as a phenotype and the genes affected in these diseases are all involved in the extracellular ATP metabolic pathway (Fig. 2*B*) (12). ATP is released from cells into extracellular space in response to physiological or pathophysiological responses, such as mechanical stress, hypoxia, and inflammation, occurring through processes like exocytosis, active transport, or cell damage (85–87). Key players are: ATP-binding cassette subfamily C member 6 (*ABCC6*), an ATP-dependent efflux transporter (88), ecto-nucleotide pyrophosphatase/phosphodiesterase 1 (*ENPP1*), a transmembrane protein that cleaves ATP to AMP and PPi, an inhibitor of mineral nucleation (58, 69, 89), and CD73, a protein encoded by ecto-5′-nucleotidase (*NT5E*), which converts AMP to adenosine (69, 89). Adenosine is a potent “retaliatory metabolite,” which helps cells respond and adapt to stress by binding to adenosine receptors or by being transported into the cell (90, 91).

Generalized arterial calcifications of infancy (GACI) is an autosomal recessive disease, often fatal, associated with mutations in the *ENPP1* (GACI type I) or *ABCC6* genes (GACI type II), respectively (92). Inactivation of these genes reduced PPi plasma concentrations in patients with GACI (93). In patients with GACI, the large muscular arteries exhibit medial dysplasia and extensive MAC, along with fragmentation of elastic fibers of connective tissues such as skin, vascular walls, and the eyes, which lead to death at an early age (94).

Pseudoxanthoma elasticum (PXE) is an autosomal recessive disorder primary caused by mutations in the *ABCC6* gene and, in some cases, *ENPP1* (95). PXE is characterized by fragmentation and severe calcification of elastic fibers in the skin, vascular walls, and eyes (96). Patients with PXE exhibit a substantial reduction in ATP and ADP concentrations, as well as a significant decrease in PPi levels (96). However, it is not clear why several individuals, including those within the same families, who has mutation in *ABCC6* gene, manifest phenotypes in infancy that overlap with those observed in patients with GACI at older ages (97). This wide spectrum of disease severity and penetrance within families is currently not understood, and the substrate of ABCC6 is not known. It is assumed that a currently unknown genetic modifier contributes to the disparate phenotypes seen in individuals with ABCC6 mutations. Because of the overlap with GACI and some PXE phenotypes, it has been hypothesized that perhaps ATP is a substrate of ABCC6 in vascular cells; however, this has currently not been proven nor disproven. Recently, Van Gils et al. (98) found a significant positive correlation between plasma PPi levels and age in patients with pseudoxanthoma elasticum (PXE) compared with healthy controls; however, PPi levels alone did not correlate with disease severity.

Disruptions in Pi homeostasis are also operative in the disease idiopathic basal ganglia calcification (IBGP), which is a rare neurodegenerative disorder caused by inactivating mutations in the phosphate transporter SLC20A2/PiT2, PDGFR1-β, or PDGF-β (99–101). A loss-of-function mutation in the *SLC20A2* gene leads to the accumulation of Pi in the ECM, whereas a mutation in the *PDGFR1*-β gene results in higher concentrations of Pi in the vessel wall and perivascular space (100). The *SLC20A2* gene encodes a type III sodium-dependent phosphate transporter that regulates the balance between sodium and Pi homeostasis (102). It is expressed in SMCs, astrocytes, and endothelial cells in the brain (102). Upon secretion from endothelial cells, PDGFB binds to its receptor, PDGFRβ, which is present on pericytes and vascular SMCs. Recently, some individuals with IBGP-causing mutations also exhibit large vessel calcification as well (103), hinting at currently unbeknownst genetic modifiers at play.

Arterial calcification due to deficiency of CD73 (ACDC), a rare genetic disease where patients present with MAC in lower extremities, is due to inactivating mutations in the *NT5E* gene, which prevent the CD73 enzyme from breaking down AMP into adenosine (69). Lack of adenosine production enhances the expression and activity of TNAP, the enzyme essential for calcification via a Forkhead box protein O1 (FOXO1) signaling axis (68, 69). The mechanistic signature of ACDC—reduced CD73 expression along with elevated levels of FOXO1 and TNAP—is also observed in popliteal arteries of non-ACDC patients exhibiting MAC (68). FOXO1 is known to regulate many cellular responses and thus its activity is tightly regulated. In control patient cells with physiological levels of CD73 on their surface, AMP is catabolized to adenosine, which activates cAMP-mediated pathways, while in ACDC patient cells, instead of cAMP pathway activation, AKT was phosphorylated (68). In general, AKT phosphorylates FOXO1 and marks it for rapid degraded by the proteosome (104). How a lack of CD73 leads to both the activation of AKT and the nuclear localization of FOXO1 is unclear; however, one detailed study provides evidence that arginine methylation is critical for FOXO1 nuclear localization and activity. Yamagata et al. (105) demonstrated that arginine methylation on FOXO1 prevents its degradation and enables FOXO1’s nuclear localization and transcriptional activator functions. The mechanism by which a lack of adenosine production promotes FOXO1 nuclear localization is inadequately understood; perhaps the answer to this gap in knowledge is related to adenosine’s role in the methionine cycle and arginine methylation.

## THE METHIONINE CYCLE

**Figure 3.**
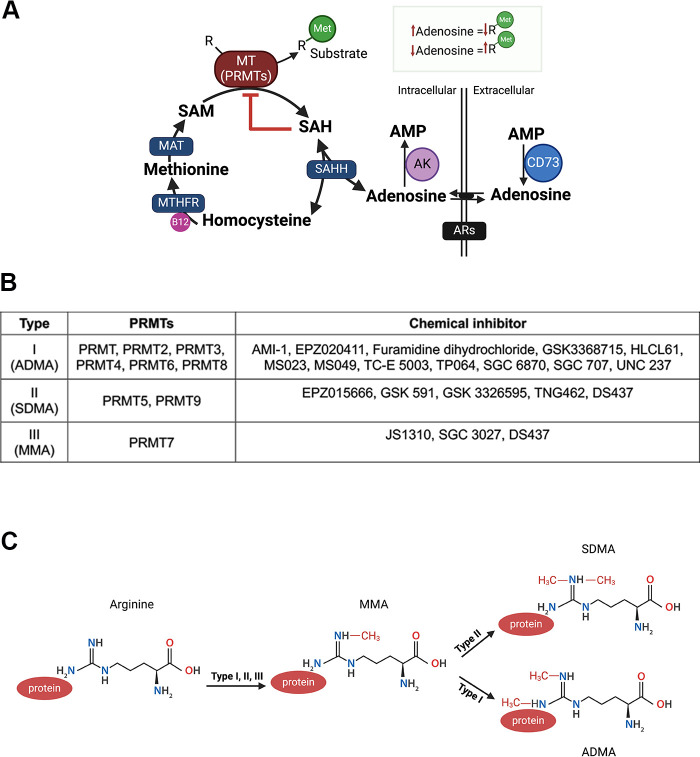
The methionine cycle and protein arginine methylation. *A*: adenosine is linked to methyltransferase activity. Extracellular adenosine monophosphate (AMP) is converted to adenosine by CD73. Adenosine binds to its receptor (ARs) or translocates into the cell. *S*-adenosylhomocysteine hydrolase (SAHH) is a bidirectional enzyme that favors condensing adenosine and homocysteine into *S*-adenocystine (SAH) and inhibits the activity of methyltransferases (MTs). 5,10 methylenetetrahydrofolate reductase (MTHFR) catalyzes homocysteine to methionine, with vitamin B12 as a cofactor. Methionine adenosyltransferase (MAT) catalyzes methionine to *S*-adenosylmethionine (SAM). *B*: list of PRMTs and their inhibitors. *C*: arginine methylation of protein by PRMTs. Type I, II, and III generate monomethyl arginine (MMA), then type II can produce symmetric dimethylarginine (SDMA) or Type I asymmetric dimethylarginine (ADMA). AK, adenylate kinase; PRMTS, protein arginine methyltransferases; R, arginine; R-Me, methylated R.

The methionine cycle, also known as the methionine metabolic pathway, is a series of biochemical reactions using the essential amino acid methionine (Fig. 3*A*). The methionine cycle metabolite *S*‐adenosylmethionine (SAM), is the methyl-donor for all protein methyltransferases (MTs). The removal of the methyl group of SAM generates *S*-adenosyl-L-homocysteine (SAH) (106, 107). Elevated SAH levels can feedback and inhibit MT activity (5). The intracellular concentrations of SAH are regulated via the bidirectional enzyme *S*-adenosyl-L-homocysteine hydrolase (SAHH). If SAH levels are high, SAHH can break down SAH into homocysteine (HCY) and adenosine, but if SAH levels are low, or if adenosine and HYC levels are high, SAHH can work in the other direction, converting HCY and adenosine in SAH (108).

As mentioned earlier, adenosine can be transported into the cell; extracellular production of adenosine can significantly impact SAHH, and thus methyltransferase activity (5). 5,10-methylenetetrahydrofolate reductase (MTHFR) is an enzyme that produces a cofactor needed for the conversion of HCY into methionine (109). The C677T polymorphism in the *MTHFR* gene correlates with an increase in plasma HCY levels and cardiovascular disease (110, 111). Meta-analysis shows that individuals with the minor alleles (TT) of the *MTHFR* gene exhibit higher HCY levels and have a significantly elevated risk of stroke compared with those with the CC genotype (112). However, the relationship between HCY levels and all-cause mortality was found to be particularly pronounced in individuals with the CC/CT genotype of the *MTHFR* gene (113).

Adjacent to the methionine cycle enzymes, adenosine kinase (AK) regulates intracellular adenosine levels by converting it to inosine. AK deficiency is a devastating developmental disease, which causes a buildup of intracellular adenosine, increasing SAH and blocking methyltransferase activity (114, 115).

## THE IMPACT OF METHIONINE CYCLE SUBSTRATES ON PROTEIN ACTIVITY

A number of methyltransferases are involved in the methylation of histones, DNA and nuclear proteins, as well as proteins related to transmembrane signal transduction, which are typically abundant in the cytoplasm (116, 117). As such, certain methyltransferases exhibit dynamic shuttling between the nucleus and cytoplasm, allowing for versatile regulation of their target substrates. This spatial distribution of methyltransferases underscores their distinct roles in different cellular compartments and highlights the complexity of their regulatory mechanisms.

### DNA Methylation

DNA methylation by DNA methyltransferases (DNMTs) is a vital for process like transcriptional regulation, genomic imprinting, and X chromosome inactivation (118–120). DNMTs are responsible for the addition of methyl group from SAM to cytosine residues of DNA, predominantly occurring at CpG dinucleotides throughout the genome, excluding CpG islands, which remain unmethylated (121). Methylated DNA sites allow for the recruitment and binding of various transcriptional repressors and activators complexes involved in histone modification and chromatin remodeling, subsequently modulating gene expression (122).

### Protein Methylation

Histones can also be subjected to various posttranslational modifications including methylation. Lysine methyltransferases (KMTs) specifically catalyze the transfer of methyl groups from SAM to lysine, whereas protein arginine methyltransferases (PRMTs) transfer methyl groups onto the guanidino nitrogen atoms of arginine (122, 123). Lysine and arginine methylation modulate the physicochemical properties of the amino acid residue, thereby influencing the structural conformation of the proteins that serve as substrates. This modification plays a crucial role in regulating protein-protein and protein-nucleic acid interactions, thus impacting various cellular processes (124). Arginine methylation is observed on diverse protein sequence motifs, with the glycine-and-arginine rich (GAR) motifs, also known as RGG boxes and RGG/RG motifs, being the most frequently reported targets of this modification (124). The RXR motif, which consists of two arginine residues separated by any amino acid, is another common substrate for PRMTs (125). The arginine amino acid contains a guanidine group consisting of five potential hydrogen bond donors positioned for potential interactions with biological hydrogen bond acceptors (126). As methyl groups are successively added to arginine residues, a hydrogen bond donor is removed, leading to structural modifications of the residue, and resulting in the methylation of various cytoplasmic and nuclear proteins (127). Arginine can undergo three forms of methylation: monomethyl arginine (MMA), asymmetric dimethylarginine (ADMA), and symmetric dimethylarginine (SDMA). PRMTs, based on their catalytic activities, are classified into three types: type I (PRMT1, PRMT2, PRMT3, PRMT4, PRMT6, and PRMT8), which convert arginine to MMA and further to ADMA (128); type II (PRMT5 and PRMT9) producing MMA before SDMA (129); and type III (PRMT7), exclusively catalyzing ADMA formation (summarized in Fig. 3*B*) (130). In blood and urine, the levels of MMA are significantly lower compared with ADMA and SDMA (131). This observation suggests that MMA, which is the initial product of protein arginine methylation catalyzed by PRMTs, undergoes immediate methylation to generate ADMA and SDMA in proteins. PRMT enzymes exhibit distinct subcellular localizations: PRMT1, PRMT9, PRMT2, PRMT4, and PRMT6 are predominantly localized in the nucleus, PRMT3 is in the cytosol, and PRMT5 and PRMT7 are primarily found in the cytoplasm (132).

The reversal of methylations is a crucial aspect of the dynamic regulation of histone modifications. In this context, the Jumonji domain-containing 6 protein (JMJD6), functioning as a demethylase enzyme, exhibits the ability to remove a methyl group from specific arginine residues on histone H3 at arginine 2 (H3R2) and histone H4 at arginine 3 (H4R3). In addition, lysine demethylases, such as KDM3A, KDM4E, and KDM5C, facilitate the demethylation of SMDR and ADMA, which result in the production of a combination of MMA and unmethylated arginine residue (133). Further contributing to the complexity of arginine modification, peptidyl arginine deiminases target multiple sites on H3 and H4. This enzymatic activity results in the conversion of protein arginine residues into citrulline through the deamination of proteins (134, 135). Overall, the impact of protein arginine methylation in calcification highlights the importance of this posttranslational modification in maintaining vascular health and homeostasis.

## THE METHIONINE CYCLE SUBSTRATES IN ATHEROSCLEROSIS

Methionine cycle substrates and protein arginine methylation have been implicated in cardiovascular diseases (Fig. 2*A*). Intimal calcification is the end result of chronic inflammatory processes and the upregulation of ROS. These processes disrupt normal vascular biology by affecting endothelial function and promoting osteogenic reprogramming of SMCs. HCY is easily auto-oxidized to produce free oxygen radicals, reduces activity of antioxidant enzymes such as glutathione peroxidase 1 and superoxide dismutase, and induces mitochondrial dysfunction, a primary source of ROS formation (136).

Hyperhomocysteinemia, characterized by elevated HCY plasma levels, is commonly correlated with age, sex, blood pressure, body mass index, lifestyle habits including nutrition, smoking, and coffee consumption (137–141). A significant positive association is observed between high serum HCY levels and various markers of cardiovascular disease, such as carotid-femoral Pulse Wave Velocity and prothrombotic factors in elderly people (142). Elevated HCY levels have long been recognized as a predictor for cardiovascular disease including atherosclerosis and myocardial infarction (138, 143–147), and correlate with the development of coronary artery and carotid vascular calcification (148–152). In individuals with coronary artery disease, a high plasma concentration of HCY is negatively correlated with high-density lipoproteins-C (HDL-C) and apolipoprotein A-I (APOA-I), which are atheroprotective markers (153). Increased levels of HCY have also been associated with peripheral arterial occlusive disease, venous thrombosis risk, and increased all-cause mortality, including both cardiovascular and noncardiovascular causes (154–159). The elevation in blood HCY concentration indirectly damages blood vessels by increasing the affinity of lipoprotein (a) for fibrin, which promotes clot formation and increases the risk of thrombosis (160). Vitamin B supplementation alone or in combination with folic acid did not decrease cardiovascular events in people with cardiovascular disease or diabetes who had high HCY plasma levels (161, 162). Elevated HCY levels in the blood serve as a critical indicator linking to an increased risk of cardiovascular disease. It is important to study how HCY affects human cardiovascular health at the cellular level and potentially inform strategies for the prevention of cardiovascular disease. As depicted in Fig. 3*A*, HCY and adenosine are metabolized by SAHH into SAH. High plasma levels of SAH were associated with an increased risk of cardiovascular events, including fatal cardiovascular diseases, nonfatal myocardial infarction, stroke, and atherosclerosis (163, 164).

Similar to the human, high serum HCY levels reduced the secreted levels of HDL-C and apoA-I and increased very low-density lipoprotein (VLDL) particles in cystathionine β-synthase-/apolipoprotein E-deficient (CBS^−/−^/apoE^−/−^) mice (153). The activity of cystathionine beta-synthase (CBS), responsible for the conversion of HCY into cystathionine, results in elevated levels of HCY and the development of more pronounced aortic lesions (165). This phenomenon is correlated with elevated plasma total cholesterol and reduced levels of HDL-C (165). HCY treatment induced the matrix remodeling via activation of matrix metalloproteinase (e.g., MMP-9) and proliferation pathway (e.g., ERK1/2), leading to the degradation of extracellular matrix in rat microvascular endothelial cells (166). These studies underscore the diverse and impactful consequences of elevated HCY levels on various pathways contributing to cardiovascular disease.

Murine studies have shown that elevated SAH levels induce the proliferation and migration of SMCs and accelerate the development of atherosclerosis in *ApoE*^−/−^ mice (167). *ApoE*-deficient mice that were subjected to a high methionine diet with B vitamin supplementation exhibited larger atherosclerotic lesion areas, and there was a negative correlation observed between elevated plasma SAH levels and the activity of DNA methyltransferase, as well as global DNA methylation activity in aortic tissue (168). This suggests that SAH may be a biomarker for atherosclerosis, and its presence may contribute to the development of atherosclerotic lesions by inhibiting DNA methylation in the aortic tissue. In *ApoE*-deficient mice, the elevation of plasma levels of SAH through the inhibition of SAHH was associated with increased atherosclerotic lesion progression, which was linked to the activation of endoplasmic reticulum stress markers (e.g., GRP78 and CHOP) (169). Increased plasma levels of SAH were negatively correlated with H3K9me3 levels at the promoters of GRP78 and CHOP genes (169). This suggest a role of SAH metabolism and histone methylation in the development of atherosclerosis. Bone marrow mesenchymal stem cells (BMSCs) from diabetic rats exhibited reduced therapeutic efficacy when compared with BMSCs to the nondiabetic rats. This reduced effectiveness was attributed to the heightened vulnerability of diabetic BMSCs to aging and their lower expression of SAHH in comparison with normal BMSCs. Notably, inhibiting SAHH weakened the beneficial effects of normal BMSCs on diabetic cardiomyopathy, while enhancing the expression of SAHH in diabetic BMSCs improved heart function, potentially by activating the Nrf2-mediated antioxidant signal (170). These findings emphasize the potential of HCY, SAHH, and SAH as a novel biomarker for assessing atherosclerotic calcification, but further research is needed to elucidate its role in MAC, which remains unclear. It is plausible that these markers are also involved in the calcification processes within the medial layer of the arterial wall; however, more research into this area is necessary to determine whether these associations are specific to atherosclerosis or applicable to all forms of cardiovascular calcification.

### The Arginine Methylation in Atherosclerosis

Arginine methylation has been observed to play a key role in regulating endothelial function, which is associated with the development of atherosclerosis. HCY is implicated in this regulatory process by leading to the uncoupling of endothelial nitric oxide synthase (eNOS), thereby reducing nitric oxide (NO) production (171). Plasma levels of ADMA serve as a potent endogenous inhibitor of nitric oxide synthase (NOS), the enzyme responsible for catalyzing the production of NO and citrulline from arginine (172). Endothelial NOS is essential for the cardiovascular system, acting as a key signaling molecule that regulates blood vessel tone, and prevents inflammation and thrombosis (173). Elevated NOS levels have been shown to independently contribute to increased risk of long-term adverse cardiovascular events in various clinical studies and in animal models (172, 174, 175). SDMA does not directly inhibit NOS activity but can compete with arginine, potentially reducing intracellular arginine availability and impairing NO production (176). In patients with cardiogenic shock, plasma metabolite analysis revealed a higher level of ADMA and decreased levels of arginine, significantly correlating with hemodynamic dysfunction and an increased risk of mortality (177). Patients with hypercholesterolemic demonstrate elevated ADMA levels and low l-arginine/ADMA ratio levels, which is associated with impaired endothelium-dependent vasodilation (172). Intravenous infusion of l-arginine in individuals with hypercholesterolemic improved the l-arginine/ADMA ratio and resulted in enhanced vasodilation (172). High plasma levels of SDMA and low levels of MMA were found to be significantly associated with obstructive coronary artery disease (178). Furthermore, elevated levels of SDMA, ADMA, and an integrated index of arginine methylation were identified as independent predictors of future major adverse cardiac events (178). These findings collectively highlight the importance of ADMA and arginine metabolism in cardiovascular health and disease.

Histone methylation regulates gene expression related to cellular function, inflammation, and lipid metabolism. By influencing these pathways, histone methylation may affect the processes leading to plaque buildup within arterial walls, thereby promoting atherosclerosis. In humans, advanced atherosclerotic lesions displayed reduced methylation of histone H3 at lysine 9 (H3K9) and histone H3 at lysine 27 (H3K27) within plaques, with H3K4 methylation correlating significantly with the severity of atherosclerosis (179). Upregulation of PRMT6, which mediates arginine methylation of H3, contributes to the development of cardiac hypertrophy in humans and mouse models (180). Related to vascular calcification, PRMT4-mediated *Opn* gene expression is elevated as a consequence of the SMC-specific reduction of the WNT signaling receptor, low-density lipoprotein receptor-related protein 6 gene (LRP6), ultimately promoting atherosclerosis (7). In atherosclerotic osteogenic reprogramming, the proinflammatory cytokine IL-6 promotes the transformation of human SMCs into osteoblast-like cells by upregulating *RUNX2* expression through the IL-6/STAT3/JMJD2B pathway, which involves alterations in histone methylation at the *RUNX2* promoter (33).

## THE METHIONINE CYCLE SUBSTRATES IN MEDIAL ARTERIAL CALCIFICATION

The role of methionine cycle substrates in MAC is an area of growing interest, yet it remains relatively underexplored compared with atherosclerotic intimal calcification. Elevated levels of HCY, a key intermediate in this cycle, have been associated with various cardiovascular diseases; however, its specific association or role in MAC pathogenesis is less well defined. The balance between HCY and its derivatives may influence the activity of enzymes and signaling pathways involved in MAC; however, more research is needed.

### The Arginine Methylation in Medial Arterial Calcification

In aortic mouse SMCs, arginine methylation of the RNA binding protein G3BP1 promotes nuclear factor of activated T-cells (NFATc4) transcription factor localization to the promoters *Opn* and *Alpl*, which promote calcification (8). Histone H3 lysine 4 demethylation (H3K4me2) editing, which controls SMCs phenotype, disrupts SMCs differentiation and contractility, impairs SMCs adaptive capabilities, and upregulates genes linked to osteochondrogenic activity, such as Runx2 and SRY-Box Transcription Factor 9 (Sox9) in rat aortic vascular SMCs (181).

PRMTs play a crucial role in modulating the function of proteins that are essential for regulating SMCs and calcification processes. PRMT3 was found to be upregulated in SMCs of medial arteries in CKD mice and is induced in in vitro calcification models. Inhibiting Prmt3 with SGC707 alleviated vascular calcification and inhibited glycolysis in CKD mice (182). PRMT3 activity promotes the differentiation of mesenchymal stem cells into osteoblasts by increasing Runx2 protein levels through asymmetric demethylation of histone H4 at Arg3 (H4R3me2a) (6). Chemical inhibition of PRMT3 has been shown to inhibit osteogenic differentiation in mice (6). *Prmt1* is highly expressed in the medial layer of both mice and human aorta, and depletion of Prmt1 in mouse SMCs resulted in impaired contractile properties, reduced expression of α-SMA, and rupture of elastin fibers (183). These effects contribute to the transition from a contractile phenotype to a synthetic phenotype (183). PRMT5 regulates SMCs proliferation and migration. Overexpression of PRMT5 induces dedifferentiation of human SMCs and suppresses the expression of contractile markers through arginine methylation of histone H3 at R8 and H4 at R3 (184). *Prmt5* expression is upregulated during osteoblast differentiation, and its inhibition suppresses NF-kB-induced osteoclast differentiation in bone marrow mononuclear cells (185). PRMT6 enhances the expression of *RUNX2*, a gene that promotes osteogenesis in mesenchymal stem cells (186).

Although SAM is a substrate for methylation, demethylation, leading to hypomethylation also impacts MAC pathogenesis. In mice, genetic insufficiency of SAHH leads to SAH accumulation, which promoted DNMT3b-mediated hypomethylation of the *H19* lncRNA, resulting in increased *Runx2* expression and activity and promoted the osteogenic reprogramming of SMCs (9); importantly, adenosine treatment suppresses *Runx2* expression in SAHH-depleted mouse SMCs, which mirrors observations seen in in vitro disease modeling of ACDC (69). SAHH deficiency led to decreased adenosine levels and a reduction in the activity of AMP-activated protein kinase (AMPK), which has been shown to exert an inhibitory effect on the expression of *Runx2* in SMCs (9). Although the data above support the roles for PRMTs, SAHH/SAH, and adenosine levels in MAC, the precise mechanism and consequences of alterations in these metabolites and enzymes remain incomplete, and the potential use of these substrates as biomarkers for MAC has not been thoroughly validated. Further research should aim to elucidate the molecular mechanisms by which PRMTs influence SMCs osteogenic reprogramming and calcification, particularly in the medial layer, and to investigate their potential impact on MAC.

### Circling Back to MAC Pathogenesis

Inactivating mutations in the *NT5E* gene that encodes CD73 protein leads to extensive MAC in the lower extremity arteries of affected patients (67, 69, 187). The lack of functional CD73 drastically reduces adenosine production/availability and may enhance PRMTs activity and activate osteogenic gene transcription, promoting calcification in part via enabling FOXO1 nuclear localization (68, 188). Exogenous treatment with TGFβ increases elastin protein production in SMCs isolated from *NT5E-*deficient mice, and elastin upregulation was associated with the activation of A2 subtypes of the adenosine receptor (37). These data support a link between CD73 and adenosine and ECM remodeling in ACDC pathology. It remains to be identified whether PRMT methylates FOXO1 and whether elevated PRMT activity on FOXO1 is controlled via reduced adenosine receptor signaling or reduced levels of intracellular adenosine. Identification of methylation of FOXO1 and other genes involved in the development of vascular calcification can be used as a marker to assess cardiovascular disease progression.

## CONCLUSIONS

There is a significant gap in understanding MAC pathogenesis and the transition of healthy cells into calcifying cells, as well as the interplay between exogenous stresses and signals and intracellular metabolism. Epigenetics and posttranslational modification of proteins is a promising area where these pathways may converge, and may help to fill in the gap between how physiological and environmental factors induce the development of diseases. The intricate interconnections among the methionine cycle, protein arginine methylation, and cardiovascular health underscore the multifaceted nature of these biochemical pathways. Insights derived from investigations into genetic disorders and clinical studies reveal that elevated levels of HCY and alterations in methyltransferase activity emerge as critical contributors to cardiovascular diseases, atherosclerosis, and vascular calcification. A comprehensive understanding of the role of methylation in vascular calcification could lead to the development of novel therapeutic strategies for preventing or treating cardiovascular disease. Further investigations are needed to fully comprehend the complex interplay between methionine metabolism, methyltransferase activity, and vascular health, with the goal of improving patient outcomes in cardiovascular medicine.

## GRANTS

This work was funded by National Heart, Lung, and Blood Institute Grant K22HL117917 and American Heart Association Grant 24POST1186619.

## DISCLOSURES

No conflicts of interest, financial or otherwise, are declared by the authors.

## AUTHOR CONTRIBUTIONS

C.S.H. conceived and designed research; P.B. and C.S.H. prepared figures; P.B. and C.S.H. drafted manuscript; P.B. and C.S.H. edited and revised manuscript; P.B. and C.S.H. approved final version of manuscript.
